# A network analysis of ICD-11 Complex PTSD, emotional processing, and dissociative experiences in the context of psychological trauma at different developmental stages

**DOI:** 10.3389/fpsyt.2024.1372620

**Published:** 2024-03-12

**Authors:** Zahra Mohammadi, Mahmood Dehghani, Fahimeh Fathali Lavasani, Hojjatollah Farahani, Ahmad Ashouri

**Affiliations:** ^1^ Department of Clinical Psychology, School of Behavioral Sciences and Mental Health (Tehran Institute of Psychiatry), Iran University of Medical Sciences, Tehran, Iran; ^2^ Department of Psychology, Tarbiat Modares University, Tehran, Iran

**Keywords:** complex PTSD, emotional processing, dissociative experiences, psychological trauma, network analysis, ICD-11

## Abstract

**Objective:**

Traumatic experiences are a significant risk factor for psychological disturbances, including disorders such as complex posttraumatic stress disorder, emotion-processing problems, and trauma-related dissociative experiences. The present investigation examined the coexistence of these symptoms using a network analysis model.

**Method:**

This study included a sample of 406 people referred to comprehensive health centers in Tehran from September to December 2023 with psychopathological syndromes. Variables were assessed using The International Trauma Questionnaire, International Measurement of Exposure to Traumatic Event checklist, Baker Emotional Processing Questionnaire, and Dissociative Experiences. A regularized partial correlation network and Glasso algorithm, in combination with Extended Bayesian information criteria, were applied to estimate the network structure.

**Results:**

Signs of unprocessed emotions and disturbance in self-organization symptoms were the most important symptoms in the symptom network, forming strong connections with other nodes. Thereby, these two symptoms can be regarded as the most important clinical manifestations in the symptom network following traumatic experiences. Three distinct symptom communities were identified: the community of traumatic experiences (childhood, adolescence, adulthood), the community of dissociative experiences (amnesia, depersonalization/derealization, and absorption), and the community of emotional processing (suppression, unpleasant emotional experience, Signs of unprocessed emotions, avoidance, and emotional control, posttraumatic stress disorder symptoms and disturbance in self-organization symptoms). The strongest edges observed were between childhood trauma-adolescence trauma (0.473) in the community of traumatic experiences, between amnesia and depersonalization/derealization (0.644) in the community of dissociative experiences, and between disturbance in self-organization symptoms and unprocessed emotions (0.324) in the community of emotional processing, indicating the recurrent occurrence of these symptoms.

**Conclusion:**

In this study, disturbance in self-organization symptoms was identified as the central psychopathologic symptom in individuals experiencing traumas at different developmental stages. It seems that adolescent trauma and not childhood trauma plays a more decisive role in the symptoms that a person manifests after traumatic experiences. Also, posttraumatic stress disorder symptoms and disturbance in self-organization symptoms were recognized in the cluster of emotional processing symptoms and can have substantial roles in prioritizing therapeutic measures.

## 1 Introduction

Prolonged, repetitive, interpersonal trauma in children (1, 2), mainly inflicted by caregivers (3, 4), with no possibility for escape due to physical, psychological, maturational, environmental, or social constraints, usually show more complex symptoms than usual PTSD injuries (1, 2). According to the 11th edition of the World Health Organization’s International Classification of Diseases (ICD-11), complex PTSD comprises six symptom clusters: the three PTSD criteria of avoidance of the trauma reminders, disturbing experiences, feelings of current threat, in addition to three disturbances of self-organization (DSO) symptoms defined as affect dysregulation (heightened emotional reactivity to minor stressors or emotional numbing), negative self-concept (such as feelings of failure or worthlessness, inadequacy, shame, and loss of self-awareness), and disturbances in relationships (such as difficulty in establishing and maintaining interpersonal relationships or avoiding relationships, isolation or exclusion) (5, 6). This disorder, which was first proposed by Judith Herman (7) and then conceptualized as complex developmental trauma by Van Der Kolk (8), refers to the unique consequences of complex traumas and maltreatment in children. Diagnostic modalities that fail to consider the history of psychological traumas and victimization in a person often lead to unsuccessful treatments (9).

Maltreatment is conceptualized as “Failure of the Average Expectable Environment.” For children, the Expectable environment includes a supportive and loving caregiver within a larger community where the child socializes. For older children, the Expectable environment consists of a supportive family, peer groups, and continuous opportunities, allowing them to be energetic (10). Early life traumas substantially hurdle the nurturing of security and trust in interpersonal relationships with attachment figures and effectively invoke coping strategies to manage and reanalyze the trauma. This phenomenon can predispose the child to the formation of insecure attachments, as a result of this diminishing the internal sense of security or external support in the face of traumas and subsequent undesirable sequences in life, paving the ground for future traumas and the development of psychological disorders (11). Abusive parents compromise their children’s ability to perceive and communicate their abstract emotional experiences. Unsuitable communication with abusive parents can lead the child to internalize these early insecure attachments, which adversely affects the development of children’s mentalizing capacities. It is believed that the relationship between insecure attachment and lack of mentalizing is maintained during adulthood, and grownups with insecure attachment are identified with insecure attachment representations to the accessibility of attachment figures. Fizke et al. found that activating insecure attachment representations could significantly reduce mentalizing capacities, causing an individual to fail to perceive others’ emotions (12). Studies on defects in the emotional processing of people with developmental trauma indicate that the severity of the trauma strongly and positively correlates with emotional awareness (the ability to perceive and pay attention to others’ emotional experiences), emotional acceptance (the ability to experience negative emotions without invoking secondary negative emotions), emotional transparency (the ability to understand others’ emotional experiences) and impulse control (the degree of behavioral control during emotional distress) (13–16).

Moreover, adults with a history of traumatic events experience intrusive memories related to the event in various forms, such as emotional numbing and attempts to avoid reminders of the trauma. In these conditions, the dissociation mechanism can act as a psychological defense mechanism for an abused child whose defense capacity has been broken by traumatic events (17, 18). Dissociation helps the person abandon such events or at least keep them far away to become emotionally unavailable (18). Although dissociation has not been defined as a core criterion of CPTSD, symptoms such as reliving the trauma in the here and now (part of the re-experiencing cluster) and emotional numbing (part of the affective dysregulation cluster) fall into this category (4, 19, 20). The relationship between dissociation and trauma is clinically significant (21). Trauma-related dissociation is a biological response to a stressful event in which the victim finds himself in a completely helpless situation, to which the body responds by stopping processing perceptual, cognitive, and emotional information (22). From an evolutionary perspective, dissociation-induced silencing may be considered an adaptive response to life-threatening conditions during which fight-and-flight responses are inadequate for survival. In such a situation, as self-defense or escape is impossible, the person can only minimize the damage by remaining silent (23). Deactivation of sensory and functional systems may occur again in the future in response to cues related to trauma and minor stressors (22). Dissociation is a cognitive avoidance mechanism that reduces intense negative emotions, such as fear, and elicits autonomic arousal (24). Dissociation also involves a significant distortion of attention by shutting down perceptual information processing, which top-down cortical-subcortical processes can mediate at the level of functional neural activity (25).

In psychopathology, clinicians are more interested in categorical rather than dimensional approaches where symptoms and signs do not occur randomly, and some change more than others. However, there are disagreements on its mechanism. The neo-Kraepelin categorical perspective states that signs and symptoms are clustered because they share a common cause. This highlights the importance of underlying and hidden pathologies. The opposite approach states that latent dimensions cause syndromic clustering. In this model, mental disorders are the main origins of disease symptoms. The network perspective is significantly different from both traditional approaches (26). The network approach to psychopathology started a decade ago with a simple hypothesis: symptoms may present as a syndrome nurtured by causal relationships between themselves (27, 28). From this perspective, symptoms are not merely the passive indicators of a “common hidden cause” but the elements of a causal system (27, 29, 30). This hypothesis has led to several theoretical, methodological, and experimental works based on the idea that mental disorders can be described as complex systems in which symptoms play an active causal role (31, 32). Therefore, latent factor models in psychopathology have been widely criticized in the last decade. In latent factor models, symptoms are considered passive receptors for common background factors independent of their environment (26). So far, atomistic approaches have been influential in psychology, in which actors are usually pictured regardless of the behaviors of other players. Therefore, such individualistic descriptions disregard context and other components (33).

The network approach is an attractive alternative for causal models in psychopathology, proposing that psychological disorders can be conceptualized as a network system consisting of interconnected elements (nodes) where changes in one node can lead to changes in other nodes or the whole network (34) In this approach, the unique/conditional connections between nodes are called edges (35). The structure of relationships between nodes and the position of nodes in the network are essential attitudinal, perceptual, and behavioral consequences for individual units and the system as a whole (33). The density of the network or the ratio of existing nodes reflects the degree to which the activation of a symptom may activate other components of the symptom network (36). A method employing centrality measures was used to examine the relative effects of these unique symptoms on the entire network, which shows that a symptom with high centrality plays an important role in the etiology and maintenance of a psychiatric disorder. This can have important therapeutic implications (37, 38). Network analysis studies conducted in this field so far have addressed the pattern of mutual relationships between CPTSD symptoms and emotional processing problems (39), anxiety and depression symptoms (40), borderline personality disorder symptoms (41), traumas in different developmental stages (42, 43) and PTSD and CPTSD symptoms (43–46). Another study investigated network relationships between cumulative trauma of childhood, CPTSD, dissociation, depression, and emotional regulation (39). The recent study only assessed the role of childhood trauma; however, we found no study on network relationships between CPTSD, emotional processing, dissociative experiences, and traumas in different developmental stages. Based on the existing research gap, our research had three main objectives: 1- Examining the role of childhood trauma vs. adolescence and adulthood traumas in the formation of the symptom network as a risk factor, 2- Evaluating symptom clustering in the network, and 3- Exploring relationships between symptoms, seeking the central symptom that interconnects various clusters, and describing the main symptoms in the network and their interrelationships.

## 2 Materials and methods

### 2.1 Participants and procedures

The present research was a descriptive-cross-sectional study conducted in comprehensive health centers (CHC) in Tehran affiliated with the Iran University of Medical Sciences from September to December 2023. The physician referred patients with psychiatric symptoms to the researcher who attended the CHC. Inclusion criteria were (1) Age between 20 and 50 years, (2) the ability to read and write in Persian, (3) the absence of psychotic symptoms, (4) the absence of bipolar disorder, and (5) the absence of brain injury and substance use disorder. People who did not meet these criteria were excluded. The individuals who expressed their willingness to participate in the research were offered to sign an informed consent form enclosing information about the study’s objectives, voluntary participation, and data confidentiality assurance. After obtaining written informed consent, the screening questions (which had already been checked by the physician in the comprehensive health centers) were again administered to ensure that participants met the study’s inclusion criteria.

Regarding inclusion criteria, 406 patients were evaluated, and their data were analyzed. This research was approved by the Ethics Committee of the Iran University of Medical Sciences (ethics code: IR.IUMS.REC.1402.454). The procedures used in this study adhere to the tenets of the Declaration of Helsinki. Informed consent was obtained from all individual participants included in the study.

### 2.2 Measure

#### 2.2.1 International trauma questionnaire

The International Trauma Questionnaire (ITQ) is an 18-item self-reported measure to assess ICD-11 PTSD and CPTSD. This questionnaire is scored on a 5-point Likert scale from 0(not at all) to 4(extremely) with a categorical approach for diagnosis and a dimensional approach for rating the severity of symptoms. For PTSD diagnosis, the individual responses to items 1 to 9 are evaluated. For CPTSD, beyond the requirement for PTSD diagnosis (i.e., items 1 to 9), the required scores related to disturbed self-organization (i.e., items 10 to 18) should also be obtained. To rate the severity of symptoms on a score range from 0 to 24, scores from questions 1 to 6 are used to assess the severity of PTSD symptoms, and those related to questions 10 to 15 are utilized to evaluate the severity of disturbed self-organization (DSO) (47). Cronbach’s alpha coefficients for internal consistency revealed values >0.77 for all subscales, except for the avoidance dimension, with a value of 0.67, indicating the acceptable validity of almost all subscales (>0.79) (48). In the present study, Cronbach’s alpha of the ITQ score was 0.93.

#### 2.2.2 International trauma exposure measure

The International Measurement of Exposure to a Traumatic Event (ITEM) checklist includes 21 life-threatening events that a person has experienced at different stages of development (childhood, adolescence, adulthood). Of these, 16 events match the definition of trauma exposure by DSM-5 (i.e., direct and indirect threats to life or sexual and physical safety), and the other five events encompass psychological traumas according to the ICD-11 trauma exposure criteria. The scoring varies depending on the objective. The total score of childhood traumas can be calculated by adding up all events before the age of 12 years, and this can be accomplished for adolescence and adulthood traumas by gathering together the events happening between 13 and 18 years old and beyond 18 years old, respectively. Finally, the overall score for traumatic events in a lifetime can be calculated by summing the scores of all events in each developmental stage. In addition, this checklist can designate the most disturbing traumatic event (i.e., the trauma index), as well as the frequency and time of its happening (49, 50).

#### 2.2.3 Emotional processing scale

Baker’s Emotional Processing Scale (EPS) is a 25-item self-reported questionnaire to assess emotional processing styles. Each item of this scale is scored on a 5-point Likert scale from 0 (completely agree) to 4 (completely disagree), measuring the five subscales of suppression, unpleasant emotional experience, signs of unprocessed emotions, avoidance, and emotional control. The total score is calculated by dividing the sum score obtained by the participant by the total number of questions, and the same method is used to calculate the score of each subscale (i.e., the total score obtained in each subscale divided by the number of its items). The EPS’s Cronbach’s alpha and retest coefficients have been reported as 0.92 and 0.79, respectively (51, 52). In Iran, EPS’s Cronbach’s alpha coefficient has been reported as 0.95 (53). In the present study, Cronbach’s alpha of the EPS score was 0.91.

#### 2.2.4 Dissociative experiences scale

The dissociative experiences scale (DES) is a 28-item self-reported scale used to evaluate depersonalization/derealization, amnesia, and absorption. This scale assesses dissociative (but not disordered) symptoms on a Likert scale from never (0) to always (100). The total score is calculated as the sum of the participants’ scores divided by the number of questions. The score of the subscales is calculated as the sum of the participants’ scores divided by the total number of questions related to each subscale. Cronbach’s alpha of the scale was reported as 0.95, and the test-retest reliability coefficient ranged from 0.84 to 0.96 (54). In Iran, Cronbach’s alpha of this scale has been reported as 0.92 (55). In the present study, Cronbach’s alpha of the DES score was 0.97.

### 2.3 Data analysis

#### 2.3.1 Descriptive statistic

At first, the basic R software package (base R) was used to describe the data. In this section, demographic variables (age, sex, education level, and marital status) and clinical variables (mean and standard deviation of the scales) were described.

#### 2.3.2 Network estimation

We estimated the network via a Graphical Gaussian Model (GGM), in which the nodes are the observed variables interconnected through non-directional edges (i.e., the connections specify no specific direction), representing partial correlation coefficients between two variables after controlling for other variables in the network. GGM estimates many parameters that likely result in some false positive edges. Therefore, it is expected to regularize GGM via the graphical LASSO. The LASSO graphic algorithm was visualized through the estimateNetwork function using the qgraph package, called by the bootnet package in R software. In this algorithm, small connections between pairs of nodes (partial correlation coefficients) reach zero, rendering an uncondensed network to a network with a condensed structure (56, 57). The Glasso algorithm is regulated using the hyperparameter gamma (g) in combination with Extended Bayesian information criteria (EBIC) (58). The hyperparameter controls the trade-off between including possible false-positive edges and removing true edges in the final network.

We chose a conservative value of g=0.5 to drive EBIC, which prefers a dense network structure with few edges. The bootnet package automatically estimates this method in qgraph using the default EBICglasso. In the resultant network, the edges between the nodes represent the conditional independent relationships between the nodes or, more specifically, the partial correlation between pairs of nodes that control the influence of all other nodes (56). We use the LASSO algorithm with the EBICglasso method for network estimation for the following reasons: 1) both the graphical LASSO algorithm and the EBICglasso method are known for their ability to control for false positives in network estimation. This is crucial in psychopathology research to ensure that the inferred connections between symptoms or psychological constructs are meaningful and not spurious (59); 2) these methods are designed to estimate the relationships between variables, assuming that certain variables are conditionally dependent on each other in the network. Understanding these conditional dependencies can gain insight into the complex interplay between symptoms and psychological processes in psychopathology (56); 3) the graphical LASSO algorithm and the EBICglasso method naturally induce sparsity in the estimated networks, which is often desirable in psychopathology research. Sparse networks can help identify the most relevant connections among symptoms or psychological constructs, leading to more interpretable and actionable insights. In simpler terms, sparse networks make understanding the relationships between different aspects of mental health easier and can provide helpful information for developing effective treatments (57). The displayed graph has specific characteristics; nodes are depicted as circles, and edges/partial correlations are shown as lines connecting the circles. Nodes that are associated with thicker edges represent stronger relationships. The direction of node connections was indicated by color; positive relationships between edges were shown in blue, and negative relationships were shown in red. Also, the size of the nodes is determined based on the frequency in the raw data, and the higher the frequency of a node, the larger its corresponding size. This layout was based on the Fruchterman–Reingold algorithm, which places the nodes with more interconnectedness near each other and toward the center of the network (60).

Exploratory graph analysis (EGA), offered by the EGAnet package in R software, was used to investigate if all three variables were members of a single community or if each formed a separate one. EGAnet uses the Louvain community detection algorithm, which can perform better than Spinglass and Walktrap. The structure of identified communities was analyzed using standardized node strength (61), which can be interpreted with the same exploratory factor analysis loading matrix method. However, community loadings are much smaller than the loadings of a classical factor analysis matrix because they show partial correlations. Effect sizes of 0.10, 0.30, and 0.50 were suggested to interpret these loadings, indicating small, medium, and large effects, respectively (62).

We estimated centrality measures in the qgraph package to identify important nodes. Depending on the definition of “importance,” each node may be considered significant from different perspectives. Different centrality measures are sensitive to relationships between a focal unit and other units. In this study, we decided to calculate the strength index and a new index of centrality, known as “expected influence,” which is more relevant and stronger for psychopathology networks than other indicators (56, 63). Expected influence (EI) is characterized by the total number of connections of a node and shows the importance of that node in the network. This is of relative importance because, even in networks with low overall edge weights, there will always be a node with a strong expected influence (64). The expected influence equals the sum of non-absolute values of edge weights/partial correlations directly connected to a node (65). They can be expressed as the active potential of the node across the network of CPTSD symptoms, dissociative symptoms, and emotional processing (26).

#### 2.3.3 Stability and accuracy analysis

Three methods were used to evaluate the accuracy and stability of the network model. First, the accuracy of edge weights was estimated by calculating confidence intervals (CIs) with the Non-parametric bootstrap method. Then, the original data set was randomly resampled to create new data sets, in which 95% CIs were calculated. Sorting the average of the bootstrapped edge weights determines whether the edge weight of the entire sample matches the bootstrap sample (56). Second, the correlation stability coefficient (CS-C) was calculated to evaluate the stability of EI using subset bootstraps (56, 66). CS-C measures the maximum proportion of cases that can be excluded so that the correlation can reach a specific value with 95% confidence. CS-C should generally not be less than 0.25 and preferably above 0.5. Third, bootstrap difference tests were performed to evaluate differences in network characteristics (67). This test was performed based on 95% CI to determine whether the centrality indices of two edge weights or two nodes are significantly different. The bootstrap method and overall stability were tested using the bootnet package.

## 3 Results

### 3.1 Sample descriptive

Out of 406 evaluated individuals, 249 (61.3%) fell into the age range of 20 to 25 years. Two hundred forty of the participants were women (59.1%). Half of the participants were single (57.6%), and 156 (38.4%) had a bachelor’s degree. The average number of experienced traumas based on the International Traumatic Event Exposure Measurement Checklist (ITEM) was 4 in childhood, 3 in adolescence, and 4 in adulthood. The mean and standard deviation of the scores of all scales were as follows: PTSD (M=8.78, SD=5.31), DSO (M=11.45, SD=6.69), EPS (M=81.82, SD=16.55), and DES (M=65.50, SD=58.06).

### 3.2 The network estimation

**Figure 1:**
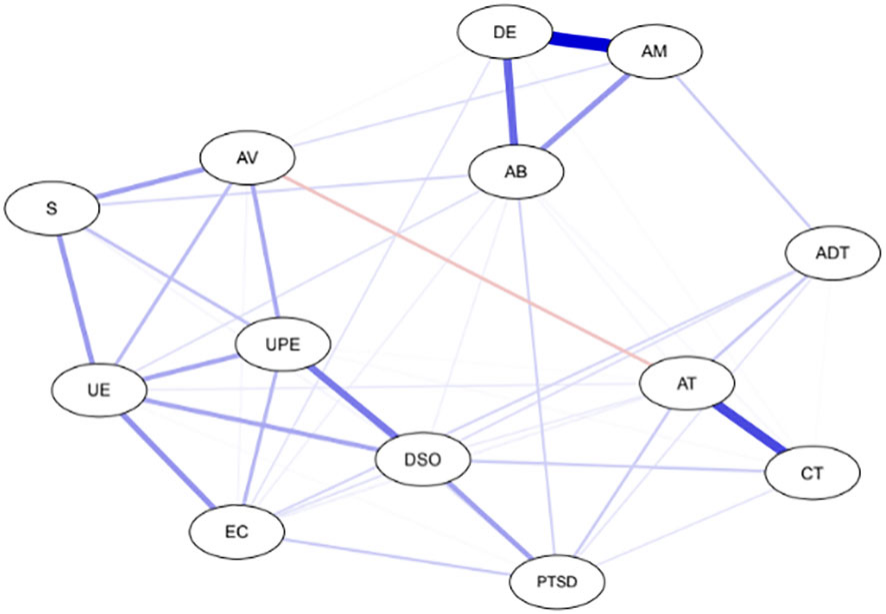
Gaussian graphical model. Nodes (circles) are scores of PTSD, DSO, EPS, DES, and traumatic events. Undirected edges, indicated by lines between nodes, represent partial correlations between variables after controlling for relationships with all other nodes. Blue lines between the circles indicate positive conditional associations; the single negative conditional association is represented as a red line (between AT and Look AV). PTSD, posttraumatic stress disorder; DSO, disturbances in self-organization; EC, Emotional Control; UE, Unpleasant Emotional Experiences; UPE, Signs of Unprocessed Emotional Symptoms; S, Suppression; AV, Avoidance; DE, Depersonalization/Derealization; AM, Amnesia; AB, Absorption; CT, Childhood trauma; AT, Adolescent trauma; ADT, Adulthood trauma.

**Figure 2:**
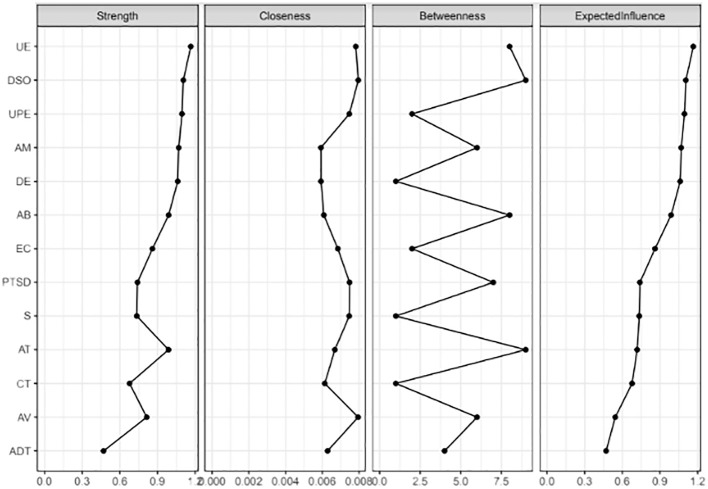
Showing centrality scores for all variables in the network.

The graphical LASSO algorithm results of network analysis of CPTSD symptoms, symptoms of dissociative experiences, emotional processing, and traumatic experiences in different developmental stages are displayed in **Figure 1**. The centrality indices showed that the most central symptoms of the network with the highest EIs included unpleasant emotional experience (EI=1.20) and DSO (EI=1.10), which had the highest EI and were, therefore, stronger than other nodes. In other words, these symptoms had a statistically significant effect on the network and played an important role in the symptoms a person experiences after traumatic experiences. Thus, the centrality estimates show an interconnected network in which UE and DSO were significant in direct and indirect paths. Also, the least important node in the network was adulthood trauma (EI=0.47) (**Figure 2**). The strongest edges identified between the nodes of dissociative amnesia and depersonalization/derealization (0.644), childhood trauma and adolescent trauma (0.473), depersonalization/derealization and absorption (0.344), and DSO and signs of unprocessed emotions (0.324). There was also a negative node between adolescent trauma and avoidance (-0.228) (**Supplementary Figure 3, Supplementary Table 1, Supplementary Material**).

The results indicated excellent accuracy and stability of the network; the correlation stability coefficient was above 0.50 (Strength=0.749, EI=0.749), which means that EI and strength were still related to the original data after removing 75% of the data (r=0.749) (**Supplementary Figures 1, 5, Supplementary Material**). The results of the non-parametric bootstrap demonstrated the lack of overlap in the 95% CIs of the bootstrap, indicating high accuracy (**Supplementary Figure 2, Supplementary Material**).

The EGA algorithm identified three distinct symptom communities, each potentially representing a different underlying structure. The first community includes three subscales of traumatic experiences (childhood, adolescence, adulthood), the second community consists of three subscales of dissociative experiences (amnesia, depersonalization or derealization, and absorption), and Finally, five subscales of emotional processing (suppression, unpleasant emotional experience, signs of unprocessed emotion symptoms, avoidance, and emotional control), PTSD, and DSO formed a separate community. The strongest edges in the communities of traumatic experiences, dissociative experiences, and emotional processing were observed between childhood traumas-adolescent traumas (0.473), amnesia-depersonalization/derealization (0.644), and DSO-unprocessed emotions (0.324), respectively, indicating the comorbidity of these symptoms. However, each symptom was directly related to at least one symptom from another community. Therefore, almost all symptoms were associated with DSO; visually, no symptoms appeared separately in the network.

## 4 Discussion

To our knowledge, our research is the first study that used a network approach to explore the interrelationships between CPTSD, emotional processing, and dissociative experiences after traumatic experiences in different developmental stages. Therefore, our study has an exploratory nature. The overall structure of the network revealed that the EBICglasso method has produced a dense network based on which we can see that childhood trauma has a strong direct relationship with adolescent trauma, adolescent trauma has more strength than childhood and adulthood trauma, and more edges connected to it. One justification for this observation may be explained by the psychoanalytic theory’s “deferred action” concept. The subject revises past traumatic experiences in the future, and it is this revision that gives them the importance and even effectiveness of pathogenic force. The memories that fall under the deferred action concept are suppressed painful traumas that manifest as symptoms (68). Another reason is that people with trauma in the early stages of life are more likely to experience revictimization in the later stages of life (adolescence and adulthood). In explaining the relationship between childhood maltreatment and revictimization in later life, psychoanalytic theory has proposed that people who have survived childhood maltreatment will unconsciously experience posttraumatic events so that they can better manage the traumatic event and its ensued occurrences, a phenomenon known as repetition compulsion (69). Other theories have explained revictimization with different mechanisms. For example, betrayal trauma theory suggests that dissociative amnesia is an underlying mechanism for revictimization (70). When a child suffers maltreatment by a caregiver, the betrayal cannot be effectively processed by avoiding the abuser’s interaction because the child needs the abusive caregiver for physical and psychological survival. Therefore, dissociation in this situation is an adaptive response that contributes to the attachment between the caregiver and the child. However, dissociation, which continues as a habit into adulthood, may interfere with information processing, including recognizing danger cues in similar interpersonal situations, leading to the risk of revictimization. Betrayal trauma theory hypothesizes that the lack of access to past information due to dissociation compromises risk recognition in future life, subsequently facilitating revictimization (71). One of the most significant results of the network was that not all symptoms were equally important in the network structure, highlighting the value of communication between nodes without emphasizing a latent underlying factor. Looking more closely at the relative importance of nodes, considering that many strong edges go to them, UE and DSO can be regarded as the most important nodes in the network. In network analysis, strength refers to the importance or influence of a node in the network, which can also be checked through weighted connections (72). When a node in the network has high strength, it can act as a bridge and communicational facilitator between different parts of the network, allowing for more control over the network flow and for the node to shape information distribution in the network, as well as its behavior and dynamics (73). Network models allow us to see how symptoms directly interact with each other in the overall network structure. So, interventions targeting the DSO and UE nodes can trigger a cascade effecting on different nodes, allowing clinicians to control dissociation and PTSD symptoms by modulating these nodes that are integrated with the emotion-processing community. Consistent with this finding, posttraumatic dissociation can be regarded as a protective mechanism against unprocessed emotions (74) in a manner that the problem in emotional processing can be considered as a node between dissociative experiences and DSO symptoms (75). These nodes can have cross effects on different types of symptoms, and considering that emotional dysregulation is one of the dimensions of DSO, it can be noted that the cluster of DSO symptoms probably plays an important role in keeping dissociative symptoms and processing problems together. This result is consistent with the results obtained from the EGA algorithm, revealing three communities of interconnected symptoms: traumatic experiences, dissociative experiences, and emotional processing. This is one of the exciting findings of the present study because PTSD and DSO fall into the emotional processing community, which could help prioritize therapeutic programs for individuals presenting with PTSD and DSO symptoms. This community displays undefined boundaries with significant connections between symptoms of different communities. Based on our findings in this research, traumatic experiences, dissociative experiences, and emotional processing can all contribute to the formation of the more general class of DSO, which is supported by previous reports (1, 20, 76).

## 5 Clinical implications

Our results show the central role of DSO and the importance of adolescent trauma in the network of symptoms. Understanding the central role of DSO in the symptom network of individuals with CPTSD highlights the importance of targeting interventions that specifically address disturbances in self-regulation, affect dysregulation, and interpersonal difficulties. Therapeutic modalities can be particularly beneficial in addressing these core features of CPTSD. Also, Psychological interventions for trauma-exposed adolescents that address DSO symptoms in addition to trauma-specific symptoms (e.g., nightmares; avoidance) may be crucial. This includes interventions to reduce risk behaviors, attachment-based interventions aimed at repairing disrupted attachment patterns, fostering secure attachments, and preventing recurrent trauma, given the negative impact of multiple traumatic events on children and adolescents.

## 6 Limitations

One of the strengths of the present study was the use of network analysis as an innovative method to create new clinical insights into CPTSD symptoms, emotional processing, and dissociative experiences after traumatic experiences in different developmental stages. The network analysis approach in psychopathology has many advantages, including 1) Network analysis provides a robust framework for exploring complex interrelationships among psychological variables, offering insights into how symptoms, behaviors, or traits influence each other within a system (35, 77, 78); 2) It provides a visual representation of relationships, facilitating the identification of central nodes, clusters, and pathways. This information can inform intervention strategies and theoretical models (35, 64, 77); 3) Network analysis can identify critical players or influential nodes in a system, which can help researchers and clinicians focus on specific targets for intervention (35, 77, 78). While network analysis is a valuable approach, past network studies of psychopathology have certain limitations that need to be addressed in future research. Most network studies in psychopathology, including our research, have used cross-sectional designs, limiting the ability to make causal inferences. Although longitudinal network studies may identify predictive relationships, causal inferences cannot be inferred from network analyses without experimental designs (37, 79). Network analyses in our research have found support for DSO symptoms being key nodes within network structures. However, these network studies have been predominantly limited to self-reported symptoms indicated by a single item, raising concerns about the reliability of symptom measurement within these networks. Moreover, network analyses have been criticized for their generalizability (i.e., the convergence of results across similar samples) (57, 80) and stability (i.e., the replicability of results across randomly selected subsamples) (79), which includes issues related to overfitting networks to particular datasets, the accuracy of parameter estimation, and the degree to which network parameters are influenced by sampling variation (57, 67, 80). In summary, while network analysis offers invaluable insights into the interplay of psychological variables, researchers must acknowledge its inherent limitations, particularly regarding data interpretation, cross-sectional designs, and causal inferences. By adopting a cautious and nuanced approach, researchers can take advantage of the strengths of network analysis while addressing potential challenges in psychological research. In addition to the limitations of network analysis, the other limitation was that this study was conducted on a non-clinical population, so it is advisable to conduct similar studies on clinical and vulnerable populations to increase the generalizability of the results.

## 7 Future directions

Despite some limitations, the findings of this study provide valuable insights into critical areas for future research. Throughout the network literature, there has been substantial discussion about the importance of highly central symptoms as prime targets for interventions with the potential to reduce psychopathology significantly. However, further research is required to assess the effectiveness of focused interventions in disrupting network structures by targeting these domains. It is crucial to acknowledge that central symptoms may be challenging to treat due to their extensive connectivity within the network, as activating any connected symptom could reactivate and maintain the central symptom. Therefore, clinicians will likely need to address central symptoms and identify and target key symptom chains and clusters in which central symptoms are embedded. Furthermore, it remains uncertain whether highly central symptoms are the most impairing aspects of psychopathology networks. In the context of our study, it may be that although DSO symptoms are highly connected within the network structure, trauma-related dissociative experiences may be more disruptive to an individual’s ‘functioning and quality of life. Additionally, due to the cross-sectional nature of the current network structures, it is still being determined to what extent nodes with high centrality temporally influence other symptoms or whether such symptoms result from other causal symptom chains. The study suggests that adapting network approaches at the individual level may provide clinicians with a more precise understanding of personal symptom relations and allow more tailored interventions. In summary, while the study identifies promising directions for future research and intervention strategies, it also underscores the need for further investigation into the complexities of central symptoms within psychopathology networks and the development of more individualized approaches to intervention.

## Data availability statement

The original contributions presented in the study are included in the article/**Supplementary Material**. Further inquiries can be directed to the corresponding author.

## Ethics statement

The studies involving humans were approved by Iran University of Medical Sciences ethics committee, Tehran, Iran. The approval number is (IR.IUMS.REC.1402.454). The studies were conducted in accordance with the local legislation and institutional requirements. Written informed consent for participation in this study was provided by the participants’ legal guardians/next of kin.

## Author contributions

ZM: Conceptualization, Investigation, Project administration, Writing – original draft. MD: Conceptualization, Supervision, Visualization, Writing – review & editing. FFL: Validation, Visualization, Writing – review & editing. HF: Data curation, Formal Analysis, Methodology, Software, Writing – review & editing. AA: Writing – review & editing.
