# Supplementary material for: A network analysis of ICD-11 Complex PTSD, emotional processing, and dissociative experiences in the context of psychological trauma at different developmental stages
Source: Front Psychiatry. 2024 Mar 12;15:1372620. doi: 10.3389/fpsyt.2024.1372620 (PMC10963615; doi:10.3389/fpsyt.2024.1372620)
Supplement: Supplementary file 1 [file DataSheet_1.docx]

Supplementary Material

# Supplementary Figures and Tables

## Supplementary Figures


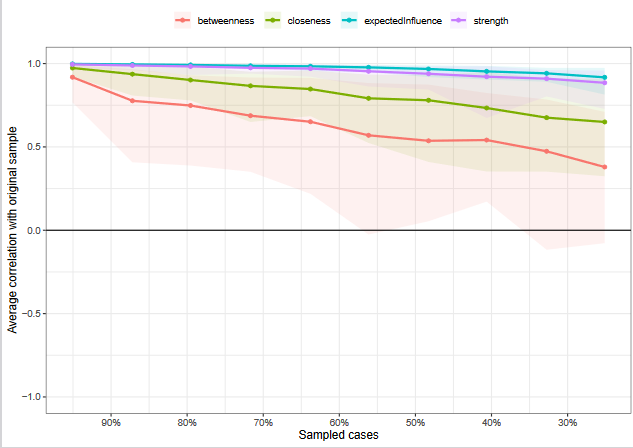


Fig 1. Stability of Centrality Estimates from Case-Dropping Bootstrap

Note. This figure shows the stability of the estimated expected influence (1EI; shown by the blue line), betweenness (red), closeness (green), and strength (pink) in the network of ICD-11 CPTSD, Psychological Trauma at Different Developmental Stages, Emotional Processing and Dissociative Experiences under a case-dropping bootstrap. This was performed with 2500 bootstrapped samples with 10 levels, dropping between 5% and 75% of participants. The x-axis shows the proportion of the sample dropped, and the y-axis represents the correlation between the original and case-dropped centrality values. The figure shows how the centrality estimates were highly stable, with the correlation with the original sample remaining very strong even after 75% of the sample had been dropped.


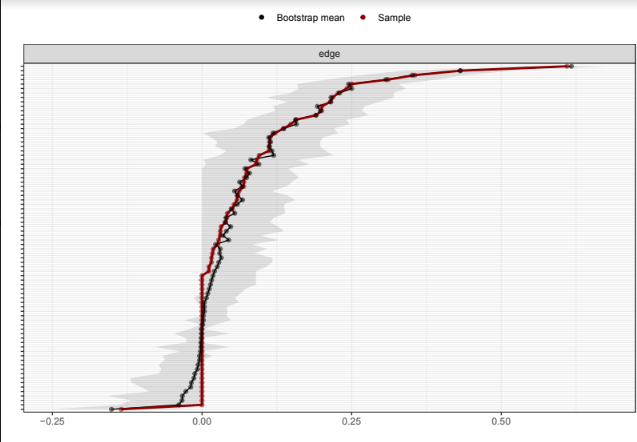


Fig 2. Accuracy of Edge Weights from Non-Parametric Bootstrap

*Note*. This figure shows the bootstrapped 95% confidence intervals (CIs) around the estimated edge weights/partial correlation coefficients in the first network of ICD-11 CPTSD, Psychological Trauma at Different Developmental Stages, Emotional Processing and Dissociative Experiences. The *x*-axis values represent the edge weights. The ticks on the *y*-axis each represent one edge (labels removed to avoid cluttering), ordered by edge weight estimated in the sample. The red line indicates the sample values (a sample value of 0 means the edge was not included in the network), whilst the black line represents the mean edge weight across 2500 bootstraps. The gray area represents the bootstrapped CIs. The narrower the CI, the more accurate the estimated edge weight.


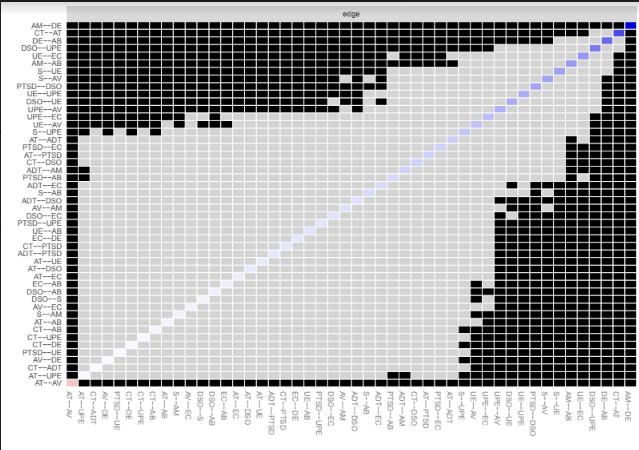


Fig 3. Significant Differences between Edges

*Note*. This figure shows the results of the bootstrapped difference tests between edge weights that were estimated to be non-zero in the first network of ICD-11 CPTSD, Psychological Trauma at Different Developmental Stages, Emotional Processing, and Dissociative Experiences. Colors in the diagonal scale with the strength of the edge weight correspond to the color of the edge in Figure 1. Black cells indicate a significant difference between the two corresponding edge weights at *p*˂.05. Gray cells indicate a non-significant difference.


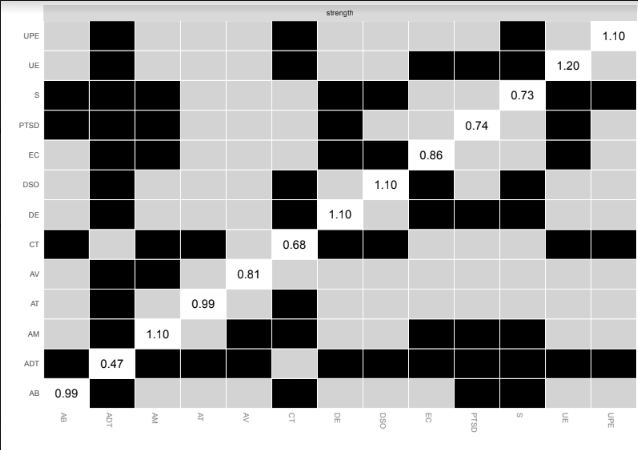


Fig 4. Significant Differences Between Strength


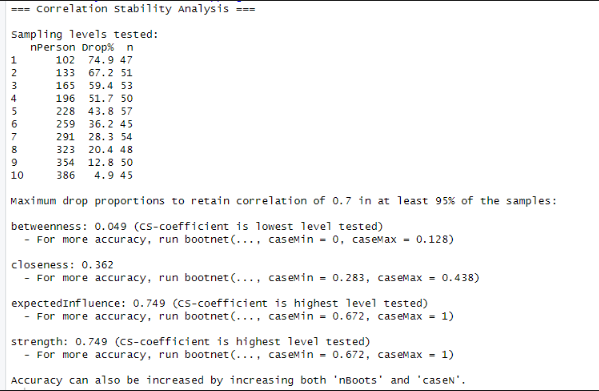


Fig 5. The correlation stability

## Supplementary Figures

| 13 | 12 | 11 | 10 | 9 | 8 | 7 | 6 | 5 | 4 | 3 | 2 | 1 | Variables |
| --- | --- | --- | --- | --- | --- | --- | --- | --- | --- | --- | --- | --- | --- |
| 0.152* | 0.069 | 0.004 | 0.065 | 0.006 | -0.106 | 0.52 | 0.069 | -.065 | -0.055 | 0.010 | 0.473** | 1 | 1. Childhood |
| 0.001 | 0.098 | 0.078 | 0.001 | -0.078 | 0.110* | -0.228** | 0.020 | 0.110* | 0.023 | 0.160* | 1 | 0.585** | 2. Teenager |
| 0.115* | 0.099 | -0.126* | -0.047 | 0.246** | 0.083 | 0.016 | -0.009 | 0.038 | -0.108* | 1 | 0.333** | 0.276** | 3. Adulthood |
| 0.103* | -0.087 | 0.120* | -0.036 | 0.077 | -0.072 | 0.237** | 0.150* | 0.244** | 1 | 0.191** | 0.195** | 0.198** | 4.Supression |
| 0.207** | 0.016 | 0.145* | -0.079 | -0.007 | 0.265** | 0.205** | 0.171** | 1 | 0.636** | 0.342** | 0.354** | 0.299** | 5.NegativeEm |
| 0.324** | 0.066 | 0.033 | -0.059 | 0.003 | 0.175** | 0.233** | 1 | 0.711** | 0.592** | 0.323** | 0.335** | 0.351** | 6.UnprocessedEm |
| -0.081 | 0.080 | -0.201** | 0.100* | 0.110* | 0.046 | 1 | 0.567** | 0.565** | 0.557** | 0.229** | 0.40 | 0.154** | 7.AvoidanceEm |
| 0.078 | 0.120* | 0.003 | 0.092 | 0.019 | 1 | 0.451** | 0.625** | 0.665** | 0.450** | 0.377** | 0.350** | 0.271** | 8.EmotionalControl |
| -0.090 | -0.131* | 0.294** | 0.644** | 1 | 0.471** | 0.439** | 0.414** | 0.459** | 0.464** | 0.395** | 0.201** | 0.263** | 9.Amnesia |
| 0.067 | 0.033 | 0.344** | 1 | 0.888** | 0.496** | 0.424** | 0.425** | 0.463** | 0.448** | 0.350** | 0.246** | 0.308** | 10.Derealization |
| 0.042 | 0.188** | 1 | 0.818** | 0.794** | 0.520** | 0.362** | 0.492** | 0.552** | 0.504** | 0.318** | 0.330** | 0.336** | 11. Absorption |
| 0.212** | 1 | 0.472** | 0.383** | 0.323** | 0.506** | 0.323** | 0.516** | 0.500** | 0.326** | 0.344** | 0.404** | 0.380** | 12. PTSD |
| 1 | 0.586** | 0.515** | 0.443** | 0.399** | 0.595** | 0.419** | 0.714** | 0.682** | 0.522** | 0.382** | 0.409** | 0.427** | 13.DOS |

Table 1. Bivariate (lower) and partial (upper) correlations of Psychological Trauma, Emotional Processing, Dissociative Experiences, and CPTSD
